# Novel PDMS-Based Sensor System for MPWM Measurements of Picoliter Volumes in Microfluidic Devices

**DOI:** 10.3390/s19224886

**Published:** 2019-11-08

**Authors:** Mihăiţă Nicolae Ardeleanu, Ileana Nicoleta Popescu, Iulian Nicolae Udroiu, Emil Mihai Diaconu, Simona Mihai, Emil Lungu, Badriyah Alhalaili, Ruxandra Vidu

**Affiliations:** 1 Faculty of Materials Engineering and Mechanics, Valahia University of Targoviste, 13 Aleea Sinaia Street, Targoviste 130004 Romania; mihai.ardeleanu@valahia.ro; 2 S.C. Celteh Mezotronic S.R.L., Calea Câmpulung Street, No. 6A, Targoviste, 130092, Romania; 3 Faculty of Electrical Engineering, Electronics and Information Technology, Valahia University of Targoviste, Targoviste 130004, Romania; iulian.udroiu@valahia.ro (I.N.U.); emil.diaconu@valahia.ro (E.M.D.); 4 The Scientific and Technological Multidisciplinary Research Institute (ICSTM-UVT), Valahia University of Targoviste, Targoviste 130004, Romania; simona.mihai@valahia.ro; 5 Faculty of Sciences and Arts, Department of Mathematics, Valahia University of Targoviste, Targoviste 130004, Romania; emil.lungu@valahia.ro; 6 Nanotechnology and Advanced Materials Program, Kuwait Institute for Scientific Research, P.O. Box 24885, Safat 13109, Kuwait; 7 Department of Electrical and Computer Engineering, University of California Davis, Davis, CA 95616 USA; 8 Faculty of Materials Science and Engineering, University Politehnica of Bucharest, Bucharest 060042, Romania

**Keywords:** microinjection sensor, PDMS capillary, micro-wire, picolitre volume measurement, MPWM microfluidic transport

## Abstract

In order for automatic microinjection to serve biomedical and genetic research, we have designed and manufactured a PDMS-based sensor with a circular section channel using the microwire molding technique. For the very precise control of microfluidic transport, we developed a microfluidic pulse width modulation system (MPWM) for automatic microinjections at a picoliter level. By adding a computer-aided detection and tracking of fluid-specific elements in the microfluidic circuit, the PDMS microchannel sensor became the basic element in the automatic control of the microinjection sensor. With the PDMS microinjection sensor, we precise measured microfluidic volumes under visual detection, assisted by very precise computer equipment (with precision below 1 μm) based on image processing. The calibration of the MPWM system was performed to increase the reproducibility of the results and to detect and measure microfluidic volumes. The novel PDMS-based sensor system for MPWM measurements of microfluidic volumes contributes to the advancement of intelligent control methods and techniques, which could lead to new developments in the design, control, and in applications of real-time intelligent sensor system control.

## 1. Introduction

The increasing demands for a complete and real-time diagnosis of a larger population and the improvement of the quality of life in general has led scientists from all over the world to study and develop multidisciplinary science and technologies [1,2,3,4,5,6]. Among them, microfluidics are based on life sciences and medical technologies of the future, which will revolutionize biology and medical diagnostics [4,5] through manipulation of very small volumes of fluids (from nano to pico and femtoliters) [1,6] and miniaturized devices [7].

To integrate one or more laboratory analyses and synthesis on a single chip of just millimeters in size, researchers and specialists have used a miniaturized device called lab-on-a-chip (LOC), which allows a quick response and diagnosis at low energy consumption and cost, using a very small volume of samples [3,8,9,10]. LOC devices, including microfluidic ones, have many advantages such as reducing the use of chemicals (reduced sample and reagent usage) that can be rare, expensive and polluting, reducing waste production, miniaturization, integration, portability, and automation [4,7,8,11]. In addition, the LOC offers a precise analysis of a large numbers of individual molecules and cells, flexibility of device design, high experimental control, and reduces measurement times [4,5,12]. Another advantage of the microfluidic cell culture device is the ability to incorporate analytical biosensors into the culture platform, thus combining, in a non-invasive way, living cells and sensors for detecting cellular physiological parameters and analyzing external stimuli in situ [12,13]. 

Nowadays, microfluidic devices can be used for biological and medical analysis, detection, control and manipulation of biological samples and cell biology research such as: analysis of the unpurified blood samples, analysis of complex mixtures and molecules (especial DNA and proteins), DNA sequencing, single cell manipulation, electrophoretic separations, drug screening, screens for protein crystallization conditions, cell culture studies and reproductive cell selection [2,9,12,14,15,16,17,18]. Also, with the help of microdevices, we can carry out environmental analysis, food analysis and control, detection and screening of residues and explosives [19,20]. Microfluidic chips provide unrivaled control over droplets and jets, which have advanced all natural sciences [21]. The development of new types of bioassay for monitoring patient response to therapy is another application [7]. LOC is a complex microsystem that includes a network of mechanical, electronic and fluid functions microchannels. These microchannels serve as pipes, valves, sensors, electronics and other structures [7,8]. LOC, bio-microelectromechanical systems (bioMEMS) and micro-total-analysis-systems (µTAS) are technologies that include both microfluidics and detection capabilities [10,22]. In microfluidic devices, the chemical and physical microenvironment can be easily controlled by using on-chip valves that allow the release of fluids containing target molecules and substances with precise timing [23]. The droplet-based microfluidic LOC platform has significant advantages for high-throughput, continuous flow and ultra-low volume studies of biological and chemical experiments [10].

For microfluidic design and fabrication it is very important to select the appropriate material that will be used and also the size and the geometry (i.e., cross-section) of the channel, and the surface characteristics of the microchannel to be made in accordance with the specific applications of the chip must be taken into account and they are briefly reviewed in the following section. 

### 1.1. Microfluidic Device Materials

Microfluidic devices can be fabricated using several materials: (a) inorganic materials such as: silicon and glass; (b) polymeric materials such as polymethylmethacrylate (PMMA), polydimethylsiloxane (PDMS) and a relative novel one, the hydrogel; and (c) Paper-based for microfluidic chips [22,24,25]. Glass is transparent, but because it is amorphous, vertical side are more difficult to etch than Si. The production of microfluidic devices made of glass or silicon is generally expensive and time-consuming [2]. Paper is a very inexpensive material and easy to work with. It is compatible with biological samples and can be chemically treated to bind to molecules or protein, but the main disadvantage when using paper–based microfluidic devices is the difficulty in detecting channels on the chip [22]. The hydrogel is a colloid made of polymeric chains of molecules spread in water. Hydrogel is very malleable, and various feature designs and sizes can be molded onto it. Furthermore, the hydrogel is commercially available, non-toxic to the cells and cost effective. One common polymer used to make hydrogel is sodium polyacrylate. Their application for microfluidic devices depends on their suitability for biological experiments. For instance, hydrogel could serve as a matrix material of new classes of solar cells and photoreactors with embedded microfluidic networks and also it is used for tissue engineering due to its high permeability and biocompatibility [25]. The most used material (both for academic and industrial applications) for prototyping and fabrication LOC device is PDMS, a mineral-organic polymer in the siloxane family. PDMS is an excellent material for the fabrication of microchannel systems/master mold because of its specific characteristics that will be discussed in detail in the next section, selection of materials for experiments [12,16]. The PDMS has some drawbacks, such as diffusivity and swelling in organic solvents [26]. The chemical structure of PDMS consist in repeating units of -OSi(CH_3_)_2_- groups, which leads to a hydrophobic surface ($\theta_{a}^{H_{2}O}=108{}^{\circ}$) [27], which is another major disadvantage of this material. There are some solutions to solve this problem, e.g., the hydrophobicity can be solved by changing the surface properties of PDMS using specific surface treatments or modification [26].

### 1.2. Techniques for Manufacturing Microfluidic Devices

Generally, microfluidic devices consist of different components, such as reservoirs, chambers, and microchannels [22]. Microchannels with varied geometries and surface properties were produced and studied over time by many researchers. There are many techniques for making microfluidic systems, all of which are consistent with their use in different fields of application [2,11,28,29,30,31,32,33,34,35,36,37]. The methods for fabrication of microfluidic devices include prototyping techniques, such as: hot embossing [29,38,39], injection molding [40] soft lithography (photolithography followed by etching and bonding) [2] rapid prototyping [2] and replica molding [16,41]. Other methods used are direct fabrication techniques like laser photoablation or laser micromachining [42] photolithography/ optical lithography [43] or photolithography followed by etching and bonding) [44] and x-ray lithography [37,45]. These techniques have advantages and disadvantages also that may or may not be used effectively in the manufacture of microchip devices. For instance, according with Qi et al. [39], the hot embossing technique has the following advantages: cost-effective, accurate and fast replication of microstructures and mass production. This technique has some disadvantages such as: (i) the restriction of applying only to thermoplastics material and (ii) the difficulty to fabricate complex 3D structures. With the micro-injection molding method [40], it is easy to manufacture complex polymer geometry, fine features and 3D geometries. This technique allows mass production, is highly automated and has a short cycle time. But has also disadvantages, i.e., the difficulty of forming large undercut geometries, high cost mold, and is applied only to thermoplastic materials, as in the case of the hot embossing technique. Another type of prototyping technique capable of fabricating 3D geometries is soft lithography, which is a cost-effective method and high resolution features (down to a few nm) can be obtained. One of the major disadvantages is the vulnerability to defects [2]. Rapid prototyping is capable of producing prototypes of almost any geometrical complexity in relatively short time [41]. The advantages of this technique compared to the methods that used the chromed mask in the photolithographic step are the reduction of time (i.e., hours instead of weeks) and reduction of costs (20–100 times more expensive than the chromatic mask that replaces the transparency. The disadvantage is the lower resolution of transparency (>20 μm) compared to the chromed mask (approx. 500 nm). The low transparency resolution leads to two walls with rough edges [2].

Replica molding has the role to form the microchannels in PDMS by generating a negative replica of a master (made by metal/hard material) in PDMS through casting of pre-polymer against the master [2]. Despite rapid and large format production, laser photo-ablation has multiple treatment sessions, and is applied for a limited number of materials [42].

Ideal for microscale features is the conventional photolithography/optical lithography but usually it requires a flat surface to start with and chemical post-treatment steps [43]. Finally, x-ray lithography allows high resolution for nano-patterns fabrication, absorption without artifacts and is capable of producing straight, smooth, walls, but imposes difficulties in the manufacturing process, consuming time and high costs [45].

#### 1.2.1. Geometry and Quality of Microchannels Surface

From the point of view of the geometry of the channels, the soft lithography technique can only make rectangular channels, which is a disadvantage in replicating the circular cross-sections of blood vessels, as well as for replicating cardiovascular flow conditions [30]. Rectangular microchannels limit cell growth on the bottom of the channel, exposing the cells to a non-physiological geometry [44]. Cells growing on the bottom of the channel have different shear stresses, which directly influence the alignment, elongation, differentiation and expression of the genes [44]. Microfabrication by photolithography method [32] is manufactured in the form of rectangular sections of channels. The rectangular channel has several disadvantages to generate drops [44,46], the fluid phase wets the upper and lower walls of the rectangular channel at a low flow rate of fluid in a continuous phase, which causes the attachments of cells to the channel walls, or even destroyed due to them: (i) surface shear force [36] and also due to (ii) capillary instability [10,47]. By increasing the flow of a continuous phase, the wetting surface of the disperse phase might be eliminated [47].

Using channels of circular section (or cylindrical geometry) instead of rectangular ones, the velocity profile can be generated evenly in the direction of the cross section, formation of stagnation areas at the corners is eliminated and a stable equilibrium position is established in the center of the channel, thus generating uniform, well controllable and monodisperse drops at a wide range of flow rates [44,47,48,49]. Another advantage of circular microchannels is that it can increase the efficiency of light transmission inside on-chip waveguides for light-sensing and light actuation methods implemented in lab-on-a-chip devices [44,50].

The surface chemistry of the microchannels is another important aspect to consider when making microchips. For example, due to its high hydrophobicity, PDMS absorbs certain organic solvents and hydrophobic analytes, causing contamination (fouling) of the inner surface channels [26]. Hydrophobic materials are difficult to fill (wet) with aqueous solutions and easily nucleate air bubbles (which makes it difficult to remove air bubbles from the channels), and consequently affect the quality of the material [2]. For this reason, to remove hydrophobicity it is necessary for PDMS to be treated with oxygen plasma to create hydrophilic PDMS surfaces by oxidation. After oxygen plasma treatment, it is necessary to maintain surfaces in contact with water or polar organic solvents [26] so as to avoid contact with the air. The best solution for surface modification is the treatment of surfaces with silanes or polyelectrolyte multilayers [27]. However, the direct contact with air results in surface rearrangements and the PDMS surface becomes hydrophobic in about 30 min [26,27].

#### 1.2.2. The Microwire-Molding Technique

Recognizing the benefits of circular microchannels, several research groups have been motivated to search for new techniques for making circular channels [44]. In this context, to improve the manufacturing techniques and to solve some of the problems or disadvantages that arise in the fabrication of microchannels, especially in the circular channels, researches developed novel, simple and fast fabrication methods. Based on pouring of liquid PDMS pre-polymer on microwires, this method is called suggestive microwire-molding [51,52,53,54,55]. The advantages of the microwire molding process are: rapidly and simply fabricate the circular-channels with perfect circular cross-sections in bulk PDMS and also the possibility to make different and/or complex topological shapes from straight channels to helical or curving micro-channel fabrication [51,52,53,54,55].

These benefits result from two main properties of PDMS: low surface energy and reversible swelling by solvents [27,52,53,54,55]. Thus, Verma et al. [55] developed for the first time a simple and cost effectiveness methods for generating straight (1D), cross-connectors (2D) and more complex (3D orientation structure) microchannels inside cross-linked PDMS blocks. They used nylon wires of varying diameters (50–250 μm) as a template to generate channels and to ease the nylon removal. Chloroform and triethylamine solvents were used for swelling the cross-linked PDMS Sylgard 184 Type, (Dow Corning, Wiesbaden, Germany) [51,52,53,54,55]. For PDMS mixtures, pre-polymer were used: curing agent for 10:1 (w/w) [51,52,53,55] and 9:1 (v/v) for [54] (Table 1).

In the same way, by embedding the microwires through molding process of PDMS mixture and then removal of microwires by swelling PDMS in different types of solvents, Jia et.al. [53], also made microchannels with different topological shapes (1D, 2D and 3D structures), in economic, flexible and convenient way in comparison with photolithography and conventional soft lithography [53]. To realize these channels, besides the casting and treatment parameters, of major importance are the preparations of microwires, substrates or supports (Table S1, Supplementary Materials).

For instance, at fabrication of straight channels [51,52,53,54,55], it is a very good stretching out the nylon/stainless steel/copper etc. microwires between rigid supports, also cleaning, drying of glass substrate/wires or degassing of PDMS mixture are required. For 2D micro-channel fabrication [53,55] it is necessary templates fabrication (2D mesh channels), formed by mechanical (hot) pressing/micro contacting and microspotting.

Thus more complex 3D structures [52,53,54,55], can be made by different techniques, such as (i) forming of a helix around a rigid cylindrical rod (Φ = 100–500 μm), heated (100 °C) to fix the helical form permanently [55], casting the PDMS mixture on 2-D nylon mesh template and curing mixture at 90 °C for 1h, or (ii) rolling up by winding around a rigid rod, casting PDMS mixture at 100 °C for 35 min [52], followed by microwire removal in different solvents mixture [52,53,55], or removal the microwire by heating and suction with vacuum pump [54]. By the technique with heating and suction removing the wires pump [54], a roughness inner surface it is obtained. Due to possible remaining of solder material after the wire removal process, additional coating treatments is necessary (i.e., wall coating of channels using PDMS solutions diluted with curing agent, in different proportions) and also corona discharge treatment to increase the hydrophilicity (for improving the fluidic flow and cell adhesion); The final step, the sterilization device by filling with 70% ethanol and washing with deionized water, make the device suitable for biological application [54].

### 1.3. The Purpose Statements

For the general purpose of generating different experiments focused on the biological environment, we present in this paper the design and fabrication by microwire molding a PDMS-based sensor with circular cross-section microchannel with a good surface quality, for the measurement of microfluidic (nanoliter to picoliter) volumes. To control the microfluidic transport at the nano-, pico- and femto-liter level, we used Microfluidic Pulse Width Modulation (MPWM) [56] system. We designed and fabricated the novel sensor especially to calibrate a microinjection MPWM system that will be used for very high precision intra-cellular insertions. The calibration of MPWM system it is necessary for reproducing with high precision and the detection and measurements of microfluidic volumes.

## 2. Materials and Methods for PDMS –Based Sensor Fabrication

### 2.1. Selection of Materials

Based on literature data [2,12,26,27,38,44,57] we selected a polydimethylsiloxane (PDMS) polymer (Sylgard 184 type) for the fabrication of a circular-cross section channel microfluidic device due to its remarkable characteristics such as: (i) excellent optical transparency, down to 280 nm [57] (ii) flexibility (i.e., it is a ductile material); (iii) elasticity (its elasticity can be “tuned” using cross-linking agents); (iv) biocompatibility (v) capacity to seals materials like glass, polystyrene and PMMA [57]; (vi) high thermal stability up to T = 300 °C [38]; (vii) permeability to gases (is more permeable to CO_2_ than to O_2_ or N_2_) [12] and also to water vapor; (viii) cost-effective production at microscale and (ix) does not require clean room environment [57]. To get a circular cross section channel we selected metallic microwires (enameled copper and steel) due the smooth surface and high flexibility of them. For creating different size of channels, we selected the following wire diameters: 63 μm and 120 μm for the enameled copper and 190 μm for the steel wire. The low surface energy and reversible swelling by solvents of the PDMS [26,27] allow the microwire removal with proper solvents. In this case, of our experiments, chloroform (with 1.39 swelling ratio) [27] were used for swelling the crosslinked PDMS. We made ultrasonic cleaning of the mold and wires in isopropyl alcohol (commercially used for degreasing and cleaning electronic components). The ultrasonic cleaning was performed with a USC-TH type ultrasonic cleaner (VWR International, LLC) at 27 °C for 30 min.

### 2.2. Fabrication Methods of PDMS Sensor

**Figure 1:**
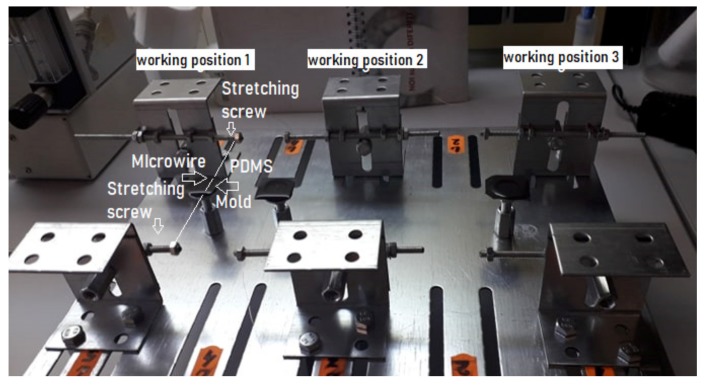
Customized stretching device with three working positions and the casted PDMS mixture inside of polymeric molds.

For fabrication of PDMS based sensor it is necessary to generate straight microchannel inside PDMS block. The main steps include: (i) Preparation of mold support and microwires, (ii) Microwire fixing and alignment, (iii) PDMS Casting, Degassing and Curing, (iv) Demolding and Microwire removal, (v) Needles insertion and sealing by needles connection to the polyethylene tubes. Preparation of molds and devices before casting: The cylindrical polymer molds were punched diametral opus, with needles (300 μm in diameter). For fixing and aligning the wires there were custom made a custom stretching device with many working positions with screw (Figure 1) respectively with magnets for attachment of the wires were made. Every position is composed of two diametric disposed elements. The stretching was performed using a microscopic system by which a linear landmark was aligned with the stretched wire. There are two stretching devices: the first one was equipped with screws and locking nuts and the second one used permanent magnets for wires fixation. At the M4 × 1 screw, we used the nut & lock-nut system to lock the convenient position to ensure the linearity of the wire. The linearity was tested with a standard edge by parallelization with the stretched wire and microscopic observations. The permanent magnets have been chosen so that by application they will secure the firm lock of the pre-tensioned wire manually. The linearity was proved as in the case with a screw.

The degassed of PDMS mixture (removing of air bubbles) was made using a low level vacuum. After casting of mixture, the casted chip made of PDMS has a height of 2.5 mm, which allows a quick natural degassing, no more than 3 h. However, if a bubble remains stuck in the PDMS table, it is mechanically removed with a needle tip. All molded chips did not show any bubbles left after casting. The degassed PDMS mixture (components A to B = 10/1 w/w) was poured in the polymer molds (Figure 1). The PDMS mixture was cured at 80 °C for 2 h and then it was cooled slowly in a Venticell type heating oven (MMM Group, Planegg, Germany), with natural air circulation,. After degassing and curing, the demoulding step consist into gentle pulled out from the PDMS block of the microwires by swelling PDMS in chloroform, leading to the formation of microchannels in the PDMS block.

The characterization of obtained microchannels using microwire-molding technique were done from microstructural point of view using different types of microscopes: (i) 1000 × 8 LED Zoom Digital USB Handheld Microscope PC Endoscope Camera Test TE306 (AmScope, Irvine, CA USA) and (ii) 40× – 800× Inverted Tissue Culture Trinocular Microscope with different video camera (custom and AmScope FMA type, Irvine, CA USA).

## 3. Volumes Detection Method

### 3.1. The Principle of Volume Detection Using PDMS-Based Sensor

By applying a constant pressure in microchannel, the flow of the liquid is defined by the microchannel geometry due to the common laws of the laminar flow [58]. The general characteristics of the liquids (incompressibility, fill and copy the shape of the vessel) [1,2], allow that the volumetric measurement of a fluid flow along the channel (with constant circular section) to be reduced to the measurement of the distance traveled between two given impulses. In this experimental work, impulses are given by a system called microfluidic pulse width modulation (MPWM). Based on a concept from the electrical engineering field, the MPWM system controls the transport of fluids through the microfluidic channels. Ardeleanu et al. [56] used MPWM to measure volumes/flow rates for future application in cell/particle trapping and release. Ainla et al. [59] used pulse width modulation (PWM) for microfluidic diluter and Unger et al. used PWM for dynamic duty cycle over time for liquid flows [60]. The experiments are performed with very small volumes of liquid using microchannels obtained by microwire molding [51,52,53,54,55] using wires of 63, 120 and 190 μm in diameter.

For precise flow control, many methods including optical volumes and flow rates measurements were developed [56,58]. An optical volumetric measurement technique has been developed to enhance the measurement in the picoliters region. Usually, a microscope with 500x magnification is used when working with picoliter volumes. The visualization of the capillary requires a very good clarity to determine the distinctive front of the transported liquid. For this reason, we used a microchannel with 120 µm in diameter, and a good magnification to have a clear image of the boundary between two immiscible fluids like air-liquid and/or liquid_1_-liquid_2_. The computerized generator of absolute coordinates needs to have a clear edge for internal accurate measurements [56,58]. Thus, in this volume determination method, the image of this boundary is very important. This generator is in fact a complex microscopic system that calculates automatically the current absolute coordinates of the mouse cursor (pointer) within the microscopic field of view. Summarizing all the above considerations, the principle of volumes detection and measurement mainly involve three elements: 1) a cylindrical capillary; 2) a fluid boundary as detectable tracking element; 3) an absolute coordinate’s generator in microscopic field of view. The capillary must be fabricated based on PDMS molding techniques [51,52,53,54,55] and it must have finally the imposed cylindrical geometry with the precise dimensions determined using microscopic tools [56,58].

**Figure 2:**
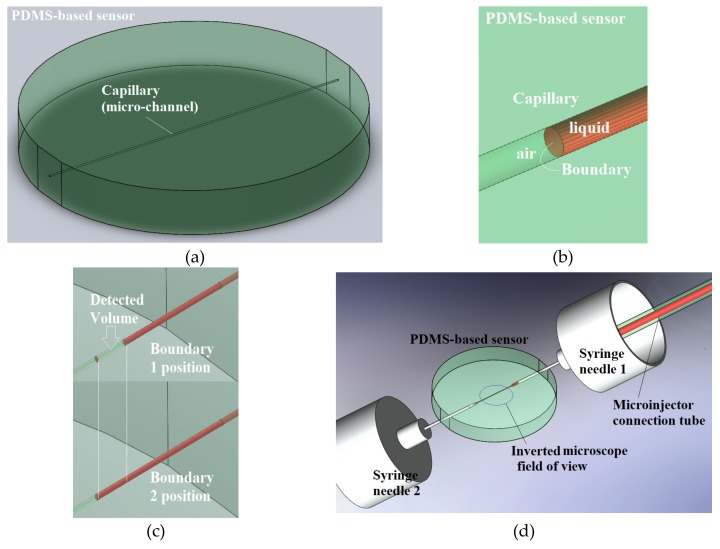
PDMS-based sensor: (**a**) Sensor-chip and (**b**) Boundary air-liquid; (**c**) Illustration of two distinct positions of successive boundaries accounted in the measurement of the microfluidic volume; (**d**) Inverted microscope field of view for PDMS-based sensor.

The PDMS chip element (Figure 2a) sustains the spatiality of the microchannel (capillary) creating the mechanical premise for the microinjector connector and inverse microscopic visualization (Figure 2d). The tracked fluid boundaries are air-liquid (Figure 2b) and the visual volume detection is obtained by computing two successive boundary positions (Figure 2c). The liquid is a commercial red color ink.

**Figure 3:**
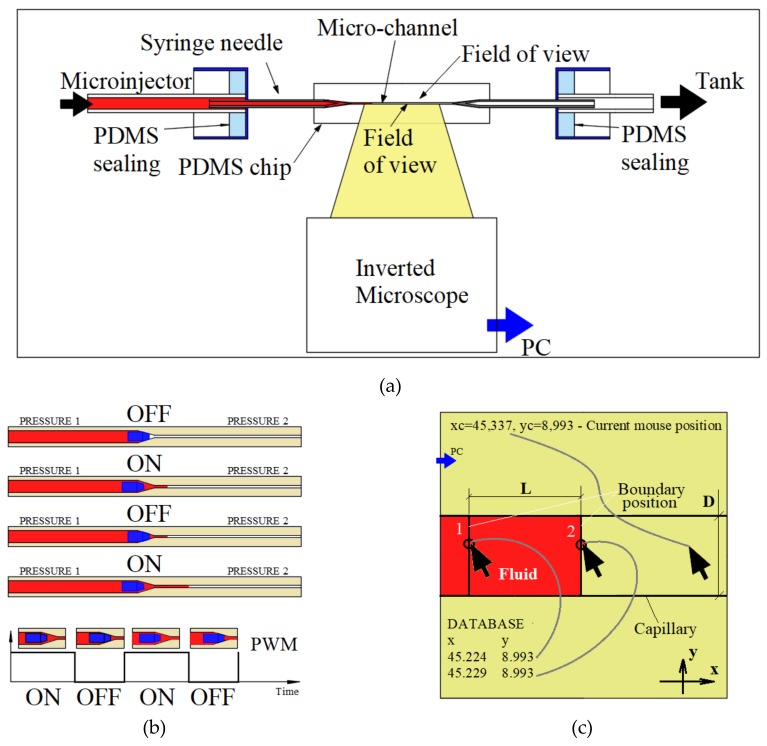
Experimental set up and measurements: (**a**) Schematic representation of the experimental set up, (**b**) MPWM principle, (**c**) The measurement performed by taking into account the advancement of the liquid boundary with the length (L) (the cross-sectional area of the channel is constant).

The PDMS-based sensor chip is connected to a microinjector system (Figure 3a). This microinjector system is a MPWM equipment type that will be used to circulate the working fluid using the pulse modulation principle (Figure 3b). The generation of fluid volumes is quantified by controlling the closing and opening times of the valve. When the valve is opened (ON position), the pressure difference between the two chambers connected by the valve will allow a precise volume of fluid to flow. When the valve is closed (OFF position), the fluid circulation is blocked. The time when a sequence of closed/open states occurs is called a period. The ratio between ON time and period is called duty cycle. The duty cycle is the basic parameter of the volume generation from the experiment, keeping the pressures and the MPWM signal frequency constant. Figure 3c shows the schematic Digital Tracking Coordinates System (DTCS) of Celteh Company (Targoviste, 130092, Romania), which allows the determination of absolute coordinates in the videomicroscope active screen. The DTCS or also called bi-dimensional micrometric terminal blocks (BMS) [61], is presented in detail in the paper of by Ardeleanu et al. [61].

The mouse pointer is the object to which DTCS interactively assigns absolute coordinates. With the “double-click” command, the current position of the mouse pointer is registered by the DTCS in a database with a certain order number. The recorded coordinates are also visible on the interface, the operator being able to use them for the purpose of determinations and calculations.

The minimum transported volume by microinjector is in the femtoliters range. In the field of view of the inverted microscope, the air-liquid boundary will be detected using DTCS (Figure 3a). Figure 3b shows the fluid advance from the initial moment at different times, t, represented by closing/opening (on/off) of the chamber with valves, controlled by the MPWM signal. The fluid advance after each MPWM signal is thus controlled and precisely determined by the advancement of very small volumes of liquid from the microfluidic level. A volume determination involves two distinct positions of the fluid boundary at two distinct moments (Figure 3b). The absolute coordinate generator has the ability to determine with high precision of less than 1 µm the current mouse cursor position. A simple click allows the position data to be recorded, in absolute coordinates of x and y. A single (pL) volume measurement involves a succession of two distinct boundary positions and two distinct position data records. The researcher uses these data sets to determine the length L of injected fluid volume by one MPWM impulse that generates the fluid movement, as shown in Figure 3b. The liquid volume determination consists in using the Equation (1):

(1) $$V=L\cdot \frac{\pi \cdot D^{2}}{4}$$

For every injected volume in capillary, the database will be populated with distinct coordinates of the position of the fluid boundary. The capillary is aligned with one of the two coordinates, which is *x*-axis in this case. The *x* becomes the main coordinate for calculating the length L of an injected volume, calculated by subtracting two successive values from the database, i.e., *x*_k-1_ and *x*_k_.

### 3.2. Method of Experimental Measurements of Picoliter Volumes

**Figure 4:**
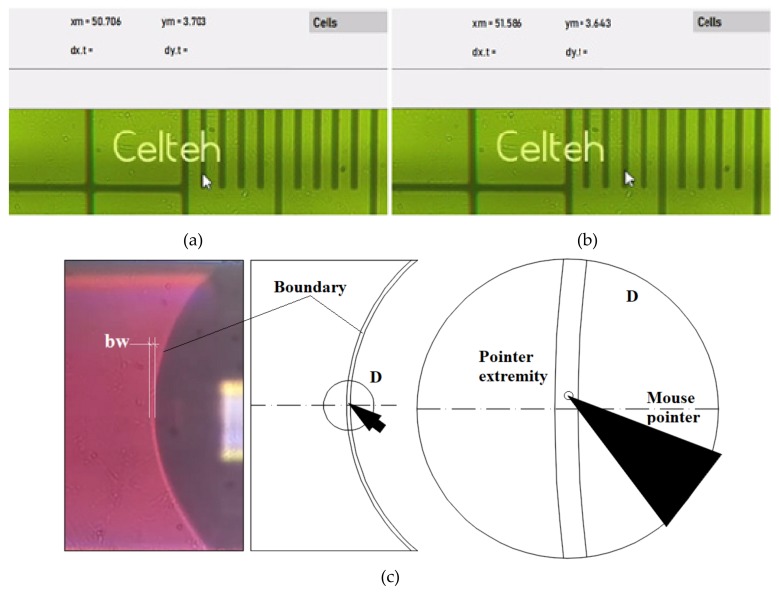
Representation of the MHT method: (**a**) microscope image capture of the first position of the mouse pointer, (**b**) microscope image capture of the second position of the mouse pointer, (**c**) schematic representation of the MHT method, applied to the liquid boundary measurement on a PDMS-based sensor

The measurement of the movement of the boundaries of liquid during fluid movements was done by two methods: the first one is the current coordinate mouse registration “mouse head tracking method” (MHT) and the second one is the “pointing method” (P). In the MHT method, the current coordinates of the mouse pointer are displayed on the interface at the associated positions on the screen. The tip of the mouse is a visible landmark that offers precision due to its small size relative to the tracked object. Figure 4 illustrates the MHT method, which was also used for MPWM calibration.

In Figure 4a,b, the mouse pointer coordinates are shown as pointed on the interface with x_m_ and y_m_. These x_m_ and y_m_ values change interactively with the change of the position of the mouse, which allows the tracking of elements of interest on the screen of the video-microscope.

**Figure 5:**
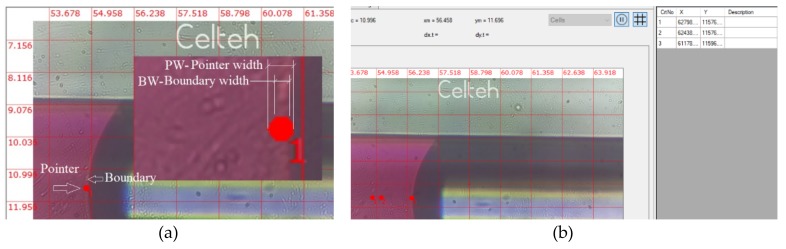
Microscope image captures of (**a**) method P for visual volumes detection (the insert shows the magnified pointer point and boundary); (**b**) Successive pointing positions.

After the calibration was performed with the MHT method, the second method, P, was used to test the fluid volume generation. For a series of successive steps of injected volume with picoliter amounts, we use the P method presented in Figure 5. We dimensioned the Pointer Width (diameter) (PW) bigger than the determined Boundary Width (BW) (Figure 5a).

In this way, the operator will position the point symmetric related to the liquid boundary in the same horizontal line (Figure 5a). The precision of measurements is determined by the operator’s scoring precision (human factor) (Figure 5b).

For calibration, we used a magnification of 800× for visualization, because the volumes of fluid passing through microchannels were of the order of 25 - 50 pL, i.e., 1 MPWM step, and this condition assumes that the distance between two successive boundaries was very small. The MHT method is appropriate in this case, because the mouse size on the working screen is quite large in relation to the thickness of the boundary and allows a good positioning of the mouse tip relative to the center of the boundary, which is visually approximated by the human operator.

For measurements, the amounts of fluid passing through microchannels are large, usually tens of MPWM steps, which means that the volumes are of the order of hundreds and thousands of pL. To include two successive boundaries on the screen, a smaller magnification (i.e., >500×) is required. In this case, the thickness of boundary is defined with fewer pixels, which leads to an increase in the uncertainty of the position of the mouse tip in the center of boundary. Therefore, a point in the shape of a red circle (marker), with a diameter slightly larger than the boundary thickness has been proposed. This marker can be identified very well if it has been positioned by the operator in the center of boundary. The tip of the mouse becomes inaccurate as a reference to boundaries, therefore the geometry of the reference object is increased, i.e., a circular point with a diameter slightly larger than the thickness of boundaries. In this way, the thickness of the boundary is positioned relative to the point, becoming very visible the symmetry of crossing the boundary through the middle of the point (red circle).

**Figure 6:**
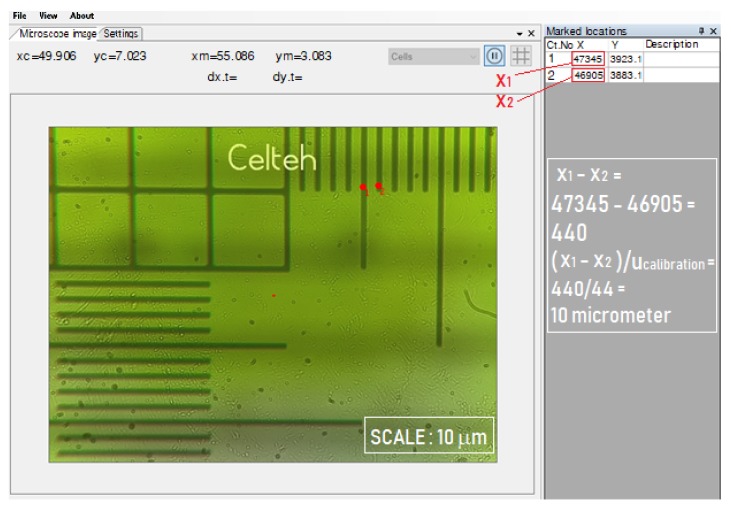
Validation of measurement precision of the distance between two successive gradation lines on the calibration scale representing 10 µm, taking into account the value of the calibration factor of 44.

To measure, the operator uses the resulted coordinates from the interface and processes these data using Equations (2) and (3). The Equation (2) is the theoretical base for Equation (3), when we took in consideration the calibration factor *u_calibration_* = 44, which results from Figure 6.

After each MPWM executed step (impulse), a new boundary position is formed in the microscope field of view. The operator must point every new position boundary that generates automatically the *X* and *Y* absolute coordinates. The microchannel is aligned with the x-axis and so it will be used to calculate only the x-coordinate from the DTCS interface table. The volume depends on the *x_k_* and *x_k_*_–1_ coordinates, which are the only variables in Equation (2) and, taking into account the following constants: *D*- microchannel diameter and *u_calibration_* – calibration DTCS factor. The volume measured at “Point *k”* is given by Equation (2):

(2) $$V_{k}=\frac{\pi \cdot D^{2}}{4}\frac{x_{k}-x_{k-1}}{u_{calibration}}10^{-3}\;[\mathrm{pL}]$$

where: *V_k_* is the volume measured at “Point k” [pL], *x_k_*_–1_ is the absolute coordinate at “Point (k-1)” [a.u.]; *_xk_* is the absolute coordinate at “Point k” [a.u.]; *D* is the microchannel diameter [µm]; *u_calibration_* is the calibration. Equation (2) is obtained from the volume calculation of a cylinder with the surface of a circle of diameter D and the length deduced from the absolute coordinates x_k–1_ and *x_k_*.

In our case, for a channel with *D* = 120 µm and *u_calibration_* = 44, the Equation (2) becomes:

(3) $$V_{k}=0.257039\cdot \left(x_{k}-x_{k-1}\right)\;[\mathrm{pL}]$$

The displacement of the liquid through the channel appears when a pressure difference is applied between the two chambers bounded by the liquid-air front. The channel has a very small section compared to the rest of the liquid path (the internal tubes of the MPWM system), which will produce a hydraulic resistance behavior. The pressures used must be appropriate to the geometry of the microchannel. The applied pressures used in these experiments were in the range of hundreds of millibars.

The volume determination will be performed automatically in the near future, through the image processing boundary recognition. At the moment, the measurement process is manual, the human factor being “the trigger” of coordinate recording, which leads to the determination of the length (L) and volume calculation. In order to generate picoliter volumes, the MPWM system must be calibrated at this working level.

## 4. Calibration of MPWM for Picolitre Volumes

The MPWM device is a complex technical system [56] that was built specifically for microfluidic transportation and measurements. An on/off valve is actuated to allow an energetic transfer of fluid between a pressurized and a depressurized space. For calibration, the pressures, frequency of the command signal and duty cycle are combined to quantify the fluidic volumes that pass through the on/off valve. Each volume generated by the MPWM system represents the quantity and the force that has an impact on the fluidic environment. In bioprocess microfluidics, extracting an adherent cell from a Petri dish requires a force applied for a short period of time, which means for MPWM a great negative pressure, high frequency and medium to high duty cycle. To transport a cell after extraction to a remote microfluidic room, speed and precision is required, this means for MPWM high pressure, high frequency and small to medium duty cycle. In this way, the MPWM signal can be tailored on specific application. For this experimental work, the final goal is to obtain a very high resolution for the 120 μm channel and a very high precision of the transported fluidic volumes. To detect the smallest volumes as possible, we used the PDMS-sensor along with the MPWM system.

On this occasion, we also tested the working capacity of the MPWM device, dimensioning the volumes generated by the MPWM system. For this purpose, we used small pressure levels, medium frequencies, and small duty cycles factor. The MPWM system presents a high repeatability of the generated volumes. The PDMS-based sensor allows to precise measuring the volumes generated by the MPWM device and storing the information along with the associated values of the input parameters. The correlations between causes (pressures, frequencies and duty cycles) and effects (generated volumes) will be the calibration process and will be stored as a map. We used in the calibration process a frequency of 100 Hz, which is one previously used for cell extraction and membrane cell elasticity testing [62].

Tables S2 and S3 (Supplementary Materials) present experimental data for the calibration in the following conditions: a constant pressure of 256 mbar, frequency of 100 Hz and duty cycle range [13.5%, 16.8%]. These tables include the *x*-coordinates measured with the DTCS system and the calculated using Equation (2) and corresponding to the determined volumes. These data allow the MPWM user to determine the calibration function. This function allows us to calculate the appropriate duty cycle parameter for the transport of certain required volumes (imposed).

**Figure 7:**
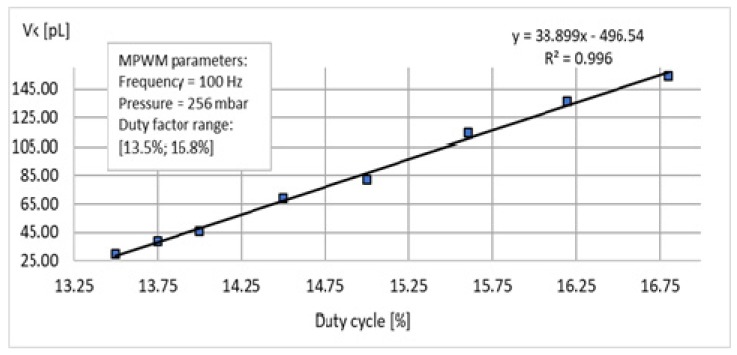
Chart of the MPWM experimental data calibration.

For data processing, the function of approximating the correlation between the generated volume size and the duty cycle was sought. For this, the average value of the volume generated by the MPWM system was calculated for each duty cycle tested. For the average values resulting from each filling factor tested, the calibration MPWM function was obtained as shown in Figure 7.

The use of this function must also take into account the deviation of measurements corresponding to each average value, deviation of measurements determined by reporting the average value at fitted values (those obtained by applying linear regression). By processing the MPWM calibration data, for each duty cycle, a series of average values of the volumes generated were obtained, which are presented in Table 2. In addition, the deviations corresponding to each average value are shown. Considering the average value as a reference, the relative deviation was calculated as the maximum fluctuation relative to the average value. The calibration function (Figure 7) is a mathematical tool for the human operator to choose the optimum MPWM parameters into a given application (transportation small/tiny volumes).

### 5. Experimental Results

#### 5.1. Microscopic Analyses of Microchannels

**Figure 8:**
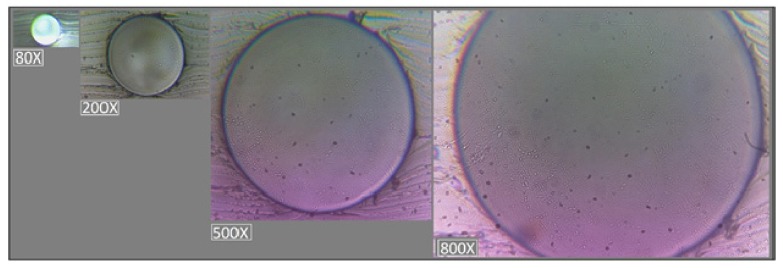
The circular cross-sectional area of microchannels produced by microwire molding technique, after 120 μm copper removal, at different magnifications (80 ×, 200 ×, 500 ×, 800 ×).

Figure 8 presents the images of the circular cross-sectional area of channels obtained after 120 μm copper wires removal. A smooth surface of microchannel was observed, which allows an uniform and well controllable flow of the fluid.

The microwire-molding techniques allow fabricating rounded channel shapes in PDMS. These fabricated channels have a circular cross section (Figure 8), that allow a very good visualization in the analyzed field, due to the efficiency of light transmission inside on-chip waveguides for light-sensing and light actuation methods.

By extracting the wire under the conditions of swelling of PDMS, the longitudinal micro-irregularities have been created that do not affect the laminar flow of the fluid, but for the precise determination of the maximum error affecting the calculation of the fluid volume, the variation of the channel section due to the roughness can be taken into account. The surface irregularities affect in a small proportion (maximum 3.8%) only the area of the channel section at the diameter level (Figure 8).

The experiments were performed by researchers from Valahia University of Targoviste in collaboration with Celteh Mezotronic. An automatic microinjection system was adapted to existing MPWM equipment, by integrating a sensor based on image processing of fluid flow through the microchannel realized with the technique known as microwire-molding. The image processing software was developed by Ardeleanu in collaboration with Company Celteh and is an innovation in the field of microscopic mechanical displacement determination with submicrometric precision.

The validation of the idea that the sensor allows the measurement of microfluidic volumes is the subject of this work.

**Figure 9:**
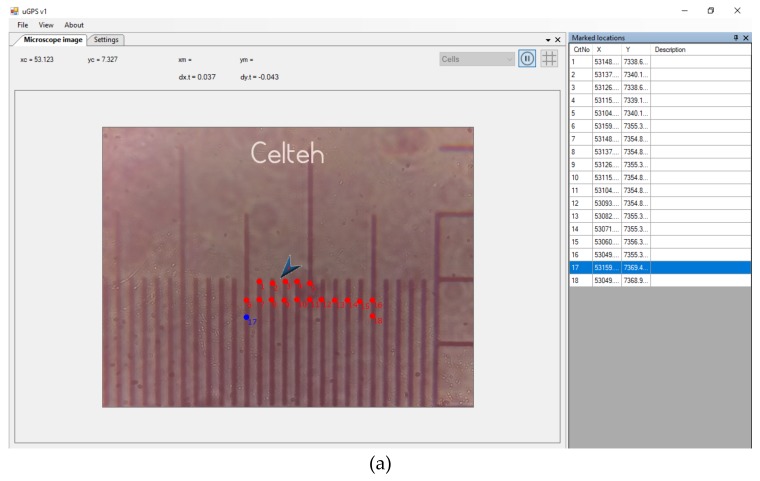

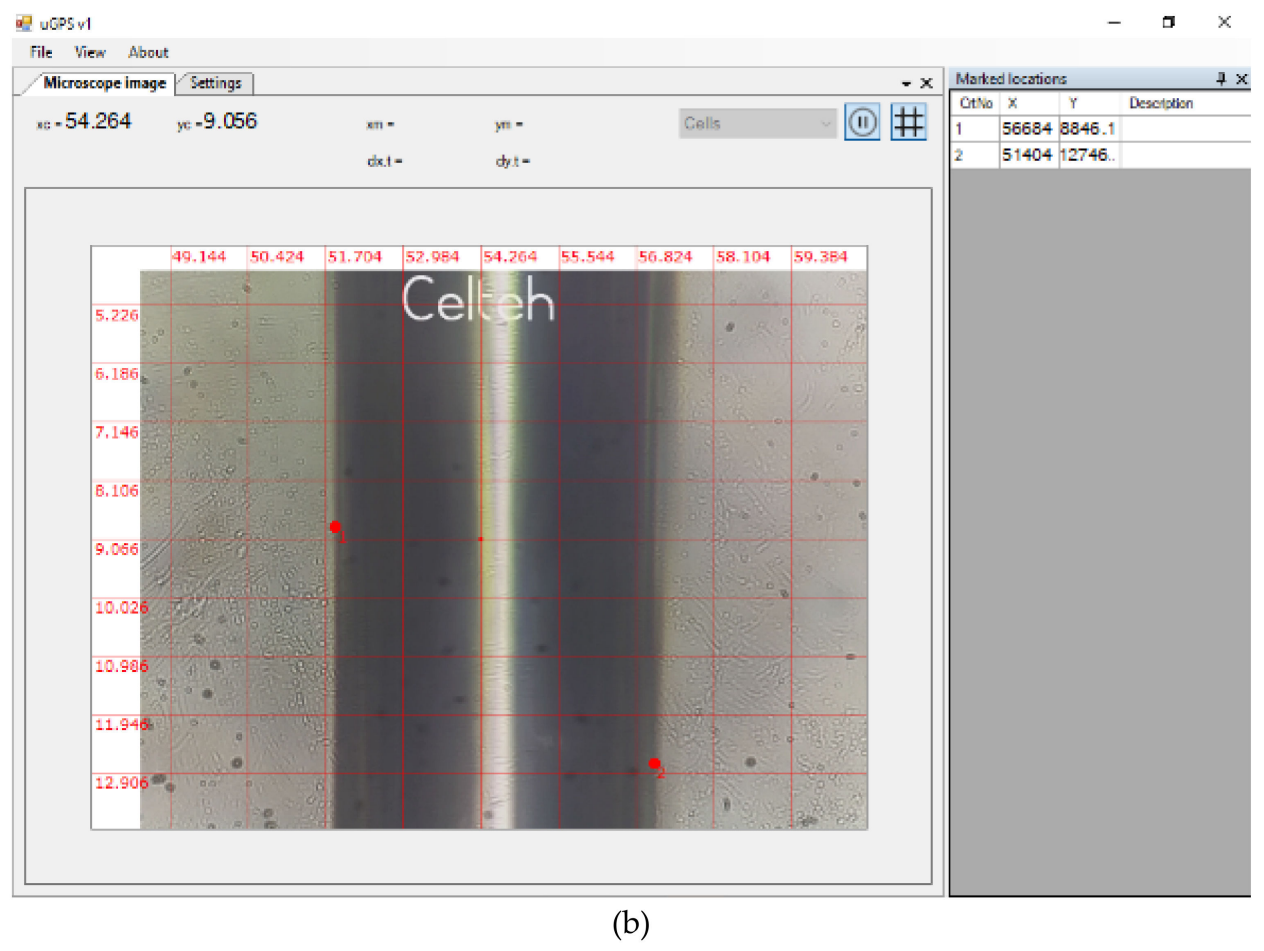
Calibration method of the DTCS system: (**a**) screen-print showing 11 red points (virtual units) representing 10 µm in length, (**b**) screen-print showing the measurement of the cannel diameter of 120 µm by DTCS.

The precision of the equipment for determining the absolute dimension is below 1 μm. The display in the interface (Figure 9) means the absolute dimensions of the mouse pointer in the field of view of the microscope, expressed in virtual numerical units.

The measurements required for the experiment represent specific distances within the field of vision, i.e., the distance between two liquid fronts generated by the MPWM signal. Figure 9 shows, for example, the calibration mode of the DTCS system. After the calibration it was found that: 11 vital points (units) represent 10 µm (Figure 9a) and thus, with this calibrated system we were able to determine the diameter of the microchannel (Figure 9b). The DTCS system can associate a number of arbitrary units to a real calibration distance (µm) so that distances of less than 1 µm can be measured with very precise approximations.

In Figure 9a calibration of 11 virtual units at a real distance of 10 μm is rendered by a specially graded ruler for this purpose. The meaning of the two red dots (1 and 2) from Figure 9b represents the positions of reaching the extremities of the visualized channel for measurement. The clarity of the image allows a precise reproduction of these edges, which leads to a precise diameter calculation. In order to determine the diameter of the microfluidic channel, a calibration of the DTCS with 44 virtual numerical units for a real distance of 10 µm was used. As can be seen in the image of the DTCS interface in Figure 9, the two reference points recorded by the human operator with the mouse on the screen, have the absolute *x* coordinates of x_1_ = 56.684 and x_2_ = 51.404. The difference between the two coordinates is the diameter of the channel expressed in virtual numerical units, i.e., 5280.

### 5.2. Results of Experimental Tests for Microfluidic Transportation

The microfluidic transportation was tested to determine the deviation of measurements obtained when small volumes were generated by the MPWM system, using the calibration function obtained previously. Using a frequency of 100 Hz, a pressure of 256 mbar and a duty cycle of 14.5%, a fixed number of MPWM steps were generated in order to obtain a given total volume. This volume is obtained by multiplying the volume corresponding to a single MPWM step by the number of steps required.

To determine the deviation of measurements due to execution, we repeated 10 times each test. Thus, the repeatability of the volumes generated by the MPWM system was obtained for a given parametric conditions. According to the calibration function, the volume for a single step under the given conditions is 67.5 pL. The four experimental tests corresponding to 5, 10, 15 and 20 MPWM steps have been generated. Table 3 and Table 4 shows the values of volumes measured in these experiments and the associated deviation of measurements (dev. of meas.) with respect to the reference volume calculated with the calibration function.

**Figure 10:**
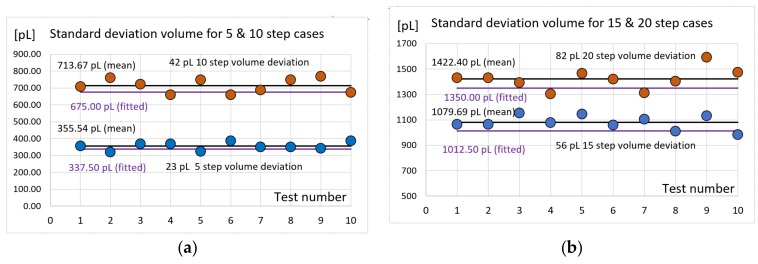
The values of the standard deviation for the four expected volumes: (**a**) for 5 and 10 steps cases; (**b**) for 15 and 20 steps cases.

The next parameter of interest is the standard deviation. In Figure 10, the values of the standard deviation for each of the four volumes tested under the same conditions 10 times are shown.

## 6. Conclusions

A PDMS-based sensor with circular cross section microchannel was obtained experimentally through the microwire molding technique. PDMS-based sensor with microchannels of 63, 120 and 190 µm were obtained and analyzed to check the quality and the micrometric sizes of the channels. The PDMS-based sensor was integrated with the MPWM system and the methodology to measure picoliter volumes was developed. We have shown that the set-up proposed in this paper was able to verify the microinjection-sensor conceptualization. The measuring process was based on absolute coordinates in the microscopic field of view with high magnification factors. The DTCS system works based on image processing, mainly detecting the mouse pointer coordinates. The human operator had the possibility to pointing with the mouse pointer, into a very precise mode, different positions of fluid boundary as main trackable element from the micro-channel, watching it on the video-microscope screen during experiments. For microfluidic volumes circulation a very high precise generator was used.

The sensor was specially designed to allow the calibration of the MPWM-injector. MPWM is based on a PWM signal that is, in fact, a “train” of identical pulses. One single pulse is a passed volume through the on/off valve. Certain applications request a given precise fluid quantity to be transported, i.e., an MPWM fluidic signal with a given number of pulses. Once the unique relation between a volume (value) and a pulse (pressure, frequency, duty cycle) is established, it only remains to generate a precise PWM signal with a precise number of pulses. In addition to the calibration of the device, the sensor serves the MPWM system to close the control loop in the microinjection process. This is why this novel PDMS-based sensor system for MPWM measurements of microfluidic (nanoliter to picoliter) volumes contributes to the advancement of intelligent control methods and techniques, and could lead to new developments in the design, control, and in applications of real-time intelligent sensor system control.

Following the experiments, the MPWM calibration function was determined, using the relationship between the microfluidic (nanoliter to picoliter) volumes and the duty cycle of the system, thus obtaining the MPWM experimental calibration diagram. Following the calibration, at 100 Hz, 14.5 duty factor and 256 mbar the microfluidic transport and the resulting picoliter volumes were tested using the pointing method. The results of the experiments confirm the quality and precision of the measurement at picoliter volumes, making it possible, in future system developments, to be applied in high-end research fields such as the automatic microinjection of biological cells.

## Figures and Tables

**Table 1:** Materials and process parameters used in the microwire molding process (PDMS, wires material and solvents type).

(Micro)wires Type	Microwires Diameters	Solvents and Proportions		Others Materials for Supporting Substrate	Ref.
Nylon	50–250 μm	Chloroform	100%	Rigid cylindrical rod of 100-500 μm Silicone oils for Channels Filling	[55]
		Chloroform + Triethylamine	70/30 (v/v)		
316 L Stainless steel	24 and 40 μm	Ethanol	100%	PMMA ancillary supporting frame and, PTFE tubes GLASS slide as substrate	[53]
		Hexane			
NiCr, Enameled Cu, Nylon	150, 350, 400 μm	Chloroform and Diisopropylamine	70/30 (v/v)	Deionized water, water and oil based colors, mineral (15 mPa.s) and silicone oils (10^3^ mPa.s) for droplet formation	[52]
Stainless steel	50, 80, 100 and 150 μm	Alcohol	100%	Particle suspensions added with 8 wt% PVP	[51]
Nylon	74, 105, 148 and 181 μm				
Soldering (H-712 type) Sn and Pb: 60/40	300 μm	-	-	Slide glass for bonding PDMS. For coating the inner walls of channels PDMS: solutions diluted with serial % of Sylgard 184	[54]

**Table 2:** A series of average values of the volumes generated by processing the MPWM calibration data, for each duty cycle.

Duty Cycle(%)	Average Volume(pL)	Fitted Value(pL)	AverageDev. of Meas. (%)
16.80	154.13	156.96	1.81
16.20	136.42	133.62	2.10
15.60	114.17	110.28	3.53
15.00	82.35	86.95	5.28
14.50	69.13	67.50	2.88
14.00	45.85	48.05	4.57
13.75	38.59	38.32	2.7
13.50	29.68	28.60	3.96

**Table 3:** MPWM repeatability experimental tests performed for 5 and 10 steps in the following conditions: frequency of 100 Hz, pressure of 256 mbar and duty cycle of 14.5%.

Measurement	5 Steps					10 Steps				
No.	Start, Virtual Unit	Stop, Virtual Unit	V_5step_ (pL)	Fitted Volume (pL)	Dev. of meas. (%)	Start, Virtual Unit	Stop, Virtual Unit	V_10step_ (pL)	Fitted Volume (pL)	Dev. of Meas. (%)
1	87754	86371	355.48	337.50	5.33	87932	85181	707.11	675.00	4.76
2	87762	86520	319.24	337.50	5.41	87928	84966	761.34	675.00	12.79
3	87744	86308	369.11	337.50	9.37	87931	85125	721.25	675.00	6.85
4	87749	86313	369.11	337.50	9.37	87940	85372	660.07	675.00	2.21
5	87758	86498	323.87	337.50	4.04	87937	85023	749.01	675.00	10.96
6	87751	86245	387.10	337.50	14.70	87923	85356	659.81	675.00	2.25
7	87757	86391	351.12	337.50	4.03	87940	85264	687.83	675.00	1.90
8	87745	86379	351.12	337.50	4.03	87626	84715	748.24	675.00	10.85
9	87750	86419	342.12	337.50	1.37	87932	84941	768.80	675.00	13.90
10	87754	86248	387.10	337.50	14.70	87946	85327	673.18	675.00	0.27

**Table 4:** MPWM repeatability experimental tests performed for 15 and 20 steps in the following conditions: frequency of 100 Hz, pressure of 256 mbar and duty cycle of 14.5%.

Measurement	15 Steps					20 Steps				
No.	Start, Virtual Unit	Stop, Virtual Unit	V_15step_ (pL)	Fitted Volume (pL)	Dev.of Meas. (%)	Start, Virtual Unit	Stop, Virtual Unit	V_20step_ (pL)	Fitted Volume (Pl)	Dev.of Meas. (%)
1	88025	83882	1064.91	1012.50	5.18	88405	82843	1429.65	1350.00	5.90
2	88085	83941	1065.17	1012.50	5.20	88449	82887	1429.65	1350.00	5.90
3	88021	83535	1153.07	1012.50	13.88	88412	82991	1393.40	1350.00	3.22
4	88015	83818	1078.79	1012.50	6.55	88394	83325	1302.93	1350.00	3.49
5	88019	83560	1146.13	1012.50	13.20	88403	82700	1465.89	1350.00	8.58
6	88035	83918	1058.23	1012.50	4.52	88396	82872	1419.88	1350.00	5.18
7	88064	83764	1105.26	1012.50	9.16	88423	83318	1312.18	1350.00	2.80
8	88059	84130	1009.90	1012.50	0.26	88452	82996	1402.40	1350.00	3.88
9	88044	83640	1132	1012.50	11.80	88405	82207	1593.12	1350.00	18.01
10	88075	84249	983.43	1012.50	2.87	88389	82651	1474.89	1350.00	9.25

## Supplementary Materials

The following are available online at https://www.mdpi.com/1424-8220/19/22/4886/s1.
