# Supplementary material for: Novel PDMS-Based Sensor System for MPWM Measurements of Picoliter Volumes in Microfluidic Devices
Source: Sensors (Basel). 2019 Nov 8;19(22):4886. doi: 10.3390/s19224886 (PMC6891790; doi:10.3390/s19224886)
Supplement: Supplementary file 1 [file sensors-19-04886-s001.pdf]

Article

Supplementary Materials: Novel PDMS-based Sensor System for MPWM Measurements of Picoliter Volumes in Microfluidic Devices

Mihăiţă Nicolae Ardeleanu [1,2], Ileana Nicoleta Popescu [1,*], Iulian Nicolae Udroiu [3], Emil Mihai Diaconu [3], Simona Mihai [4], Emil Lungu [5], Badriyah Alhalaili [6] and Ruxandra Vidu [7,8,*]

Table 2. Straight channels fabrication of 1D[1], 2D[2] or 3D[3] microchannel fabrication in PDMS.

Preparation of Microwires, Substrate or Supports	Curing Parameters	Microwire Removal and /or Others Processing Steps	Ref.
Stretching out nylon between two rigid supports; Spreading Nylon in a pool of PDMS liquid	90 °C for 1h	Nylon wires removal: Swelling PDMS in pool of $CHCl_3$ (PDMS swell 15% by length). De-swelling by drying at 25 °C in atm.	[55][1]
Templates fabrication: Hot pressed (100 °C) of nylon (at normal directions); casting the PDMS on a 250 µm channel		Swelling PDMS network templates with 70/30 $CHCl_3$ and Et3N solvent (PDMS swell: 25-35% by length); 1st Drawn Out the 50 µm, wire and 2nd the 250 µm wire	[52][2]
The nylon was spun in the form of a helix around a rigid rod (Φ=100-500 µm, heated (100 °C) wire and rod.		Immersion of the helix in $CHCl_3$ and Et3N (70:30 v/v) solvent (30-40% (by length swelling of the PDMS.). 5 g load was to remove wire.	55[3]
Glass substrat and wires cleaning [4,5] Aligning the wires on glass slide /PMMA supporting frame	60 °C for 2 h	Swelling PDMS by immersion for 2-8 h into ethanol pool (swelling coefficient 1.04); Drown Out wires from the PDMS	53[1]
Channel intersection formed by mechanical pressing/micro contacting and microspotting		Immersion for 6 h into alcohol (using ultrasonic oscillation), for swelling, pulled out the wires from the PDMS block	53[2]
Glass substrate and wires cleaning [4,5] Curving and aligning wire		Immersion it in hexane for 4-8 h. Carefully drew out the microwire	53[3]
Degassing PDMS mixture; Aligning the wires and stretching its inside the semicylindrical mold cavity	100 °C for 35 min	Swelling PDMS by immersion in a 70/30 (v/v) of Chloroform and Diisopropylamine	52[1]
Degassing PDMS mixture. Winding around a rigid rod		Pulling Off the WIRES from the PDMS. Droplet Formation	52[3]
Isopropanol cleaning and DI water; dried with nitrogen gas; aligned microwires; fixed in a mold	80 °C for 2 h	PDMS Cutting; Swelling PDMS (6 h into alcohol, with ultrasonic oscillation); Pulled Out the wires; Punching orifices using a needle; Sealing.	51[1]
Winding the soldering wire on an inflexible base rod (iron pin) with a curvature radius of 300 µm for making helical or double-helical structures	100 °C for 1 h	Metal wire removed by heating in convective oven with vacuum pump; Vacuum pump suction: 45 l/min, T = 190 °C. PDMS solutions diluted with curing agent for coating channels, surface treatments[6]	54[3]

[1] Fabrication the straight channels by embedding wire inside the PDMS [51, 53, 55]; [2] Fabrication the cross channel (2D structures) [53]; [3] Fabrication of helical [52-55] or curving [53] micro-channels.[4] Glass substrate and wires cleaning: O_2 plasma t = 5 min, pO_2 = 0.1 MPa, power 30 W [53]; [5] Ultrasonic cleaning in acetone, ethanol and Millipore pure water = 10 min, dried by N_2 [53]; [6] Corona discharge treatment to increase the hydrophilicity; sterilization (with 70% ethanol), DI washing [54].

Commented [M1]: Please consider this suggested change and please format all the references in the table in that way.

Table 3. MPWM calibration data, for duty cycle of 13.5–14.5 %, at 100 Hz and 256 mbar.

Duty Cycle											
13.50%			13.75%			14.00%			14.50%		
X_{k-1} Virtual Unit	X_k Virtual Unit	V_k (pL)	X_{k-1} Virtual Unit	X_k Virtual Unit	V_k (pL)	X_{k-1} Virtual Unit	X_k Virtual Unit	V_k (pL)	X_{k-1} Virtual Unit	X_k Virtual Unit	V_k (pL)
83890	84010	30.8	83897	84042	37.27	83860	84042	46.78	82811	83078	68.63
83779	83890	28.5	83739	83897	40.61	83683	83860	45.5	82550	82811	67.09
83668	83779	28.5	83586	83739	39.33	83511	83683	44.21	82284	82550	68.37
83549	83668	30.6	83437	83586	38.3	83334	83511	45.5	82023	82284	67.09
83438	83549	28.5	83288	83437	38.3	83162	83334	44.21	81744	82023	71.71
83323	83438	29.6	83144	83288	37.01	82985	83162	45.5	81466	81744	71.46
83208	83323	29.6	82995	83144	38.3	82798	82985	48.07	81205	81466	67.09
83097	83208	28.5	82837	82995	40.61	82627	82798	43.95	80944	81205	67.09
82986	83097	28.5	82683	82837	39.58	82445	82627	46.78	80666	80944	71.46
82863	82986	31.6	82539	82683	37.01	82263	82445	46.78	80405	80666	67.09
82739	82863	31.8	82385	82539	39.58	82086	82263	45.5	80145	80405	66.83
82624	82739	29.5	82232	82385	39.33	81899	82086	48.07	79866	80145	71.71
82509	82624	29.5	82074	82232	40.61	81722	81899	45.5	79600	79866	68.37
82398	82509	28.5	81930	82074	37.01	81536	81722	47.81	79339	79600	67.09
82283	82398	29.5	81785	81930	37.27	81354	81536	46.78	79072	79339	68.63
82172	82283	28.5	81627	81785	40.61	81182	81354	44.21	78800	79072	69.91
82057	82172	29.5	81478	81627	38.3	81010	81182	44.21	78527	78800	70.17
81934	82057	31.6	81329	81478	38.3	80823	81010	48.07	78255	78527	69.91
81823	81934	28.5	81185	81329	37.01	80646	80823	45.5	77976	78255	71.71
81703	81823	30.8	81041	81185	37.01	80464	80646	46.78	77715	77976	67.09
81588	81703	29.6	80892	81041	38.29	80293	80464	43.95	77437	77715	71.46
81469	81588	30.9	80743	80892	38.3	80116	80293	45.5	77165	77437	69.91
81354	81469	29.6	80589	80743	39.58	79939	80116	45.5	76892	77165	70.17

Table 4. MPWM calibration data, for duty cycle of 15-16.8 %, at 100 Hz and 256 mbar.

Duty Cycle											
13.50%			13.75%			14.00%			14.50%		
X_{k-1} Virtual Unit	X_k Virtual Unit	V_k (pL)	X_{k-1} Virtual Unit	X_k Virtual Unit	V_k (pL)	X_{k-1} Virtual Unit	X_k Virtual Unit	V_k (pL)	X_{k-1} Virtual Unit	X_k Virtual Unit	V_k (pL)
83612	83932	82.2	85719	86164	114.4	85312	85836	134.7	86519	87113	152.7
83293	83612	82	85275	85719	114.1	84775	85312	138	85914	86519	155.5
82968	83293	83.5	84834	85275	113.4	84247	84775	135.7	85315	85914	154
82637	82968	85.1	84390	84834	114.1	83715	84247	136.7	84721	85315	152.7
82323	82637	80.7	83949	84390	113.4	83191	83715	134.7	84127	84721	152.7
82004	82323	82	83504	83949	114.4	82663	83191	135.7	83517	84127	156.8
81679	82004	83.5	83064	83504	113.1	82131	82663	136.7	82913	83517	155.3
81365	81679	80.7	82623	83064	113.4	81599	82131	136.7	82313	82913	154.2
81035	81365	84.8	82185	82623	112.6	81062	81599	138	81719	82313	152.7
80715	81035	82.2	81737	82185	115.2	80525	81062	138	81125	81719	152.7
80396	80715	82.0	81289	81737	115.2	79997	80525	135.7	80531	81125	152.7
80076	80396	82.3	80845	81289	114.1	79473	79997	134.7	79927	80531	155.3
79762	80076	80.7	80397	80845	115.2	78937	79473	137.8	79322	79927	155.5
79438	79762	83.3	79953	80397	114.1	78404	78937	137	78728	79322	152.7
79124	79438	80.7	79505	79953	115.2	77872	78404	136.7	78129	78728	154
78799	79124	83.5	79057	79505	115.2	77344	77872	135.7	77519	78129	156.8
78485	78799	80.7	-	-	-	-	-	-	-	-	-
78171	78485	80.7	-	-	-	-	-	-	-	-	-
77851	78171	82.3	-	-	-	-	-	-	-	-	-

77526	77851	83.5	-	-	-	-	-	-	-	-	-
77196	77526	84.8	-	-	-	-	-	-	-	-	-
76877	77196	82.0	-	-	-	-	-	-	-	-	-
76563	76877	80.7	-	-	-	-	-	-	-	-	-
